# Supplementary figures and images for: Bactabolize is a tool for high-throughput generation of bacterial strain-specific metabolic models
Source: eLife. 2023 Oct 10;12:RP87406. doi: 10.7554/eLife.87406 (PMC10564454; doi:10.7554/eLife.87406)

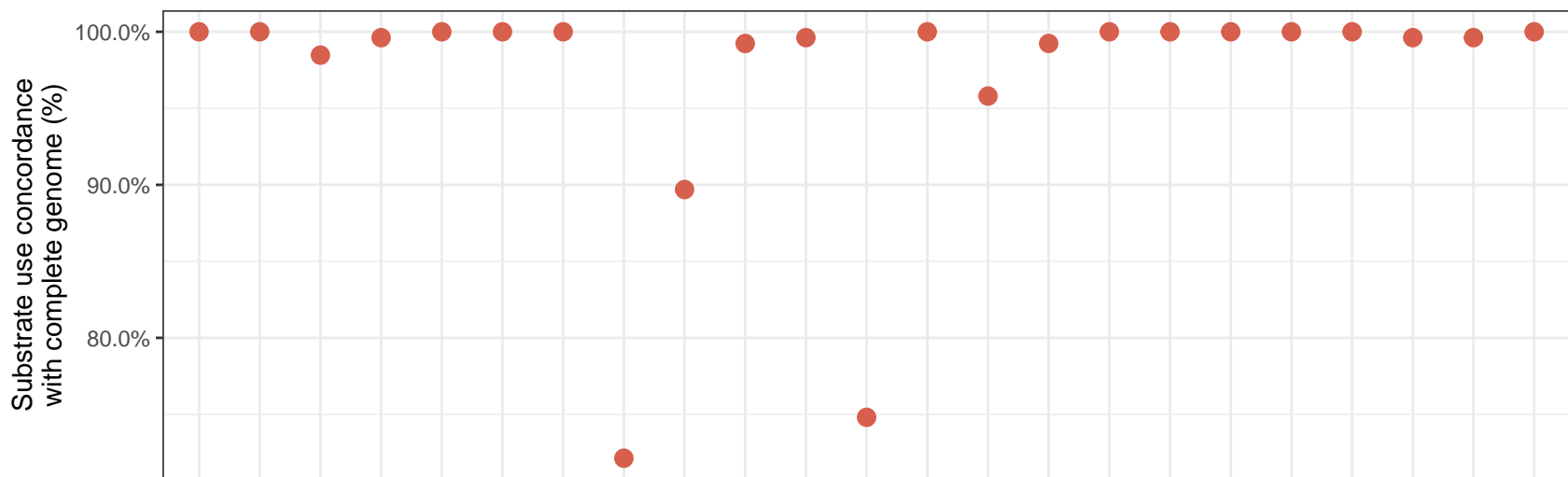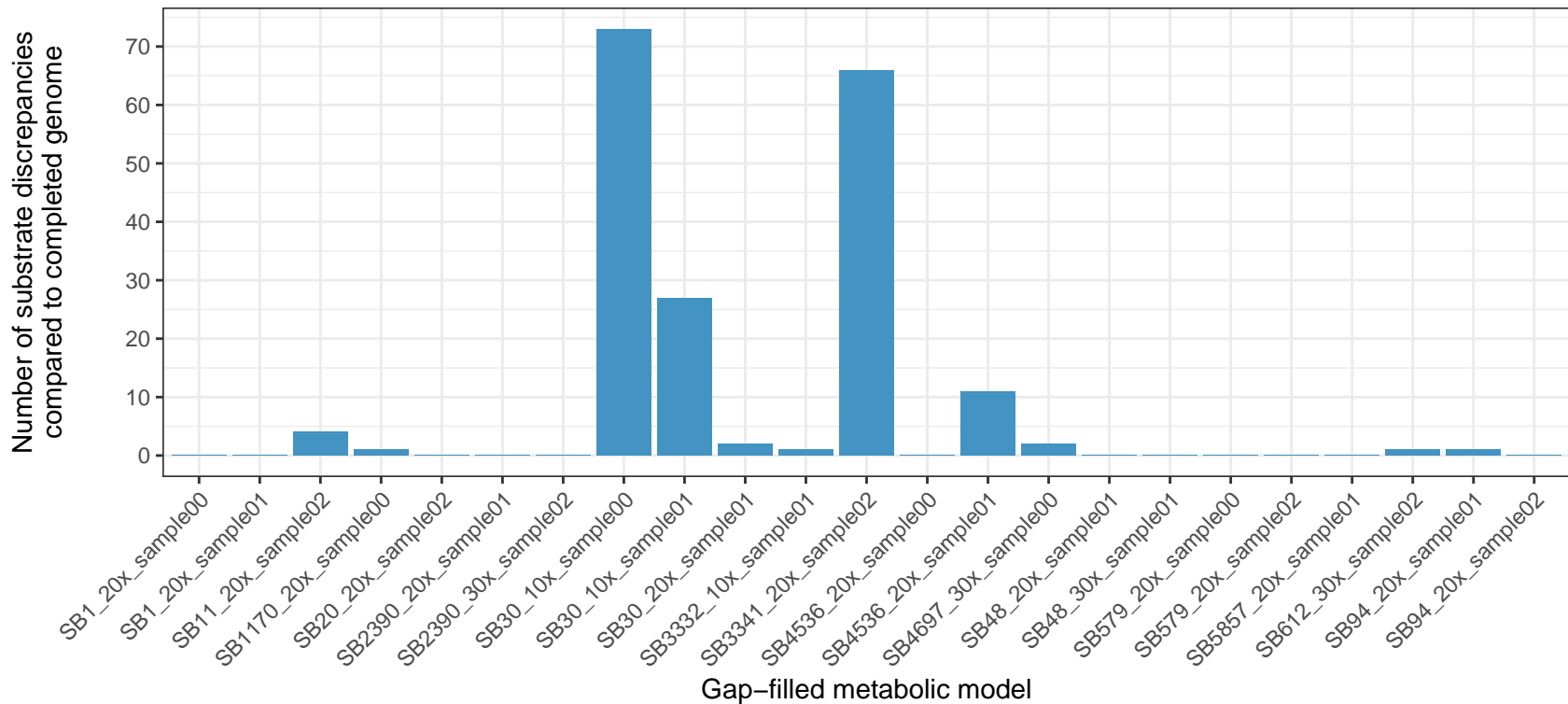

Supplement: Supplementary file 1. — The dots indicate percentage concordance with the completed genome model, while the columns indicate number of substrates with discrepancies (no simulated growth in patched model, but growth in completed genome model). [file elife-87406-supp1.pdf]
